# Spaghetti Enriched with Inulin: Effect of Polymerization Degree on Quality Traits and α-Amylase Inhibition

**DOI:** 10.3390/molecules27082482

**Published:** 2022-04-12

**Authors:** Nunzio Cardullo, Vera Muccilli, Vita Di Stefano, Sonia Bonacci, Lucia Sollima, Maria Grazia Melilli

**Affiliations:** 1 Department of Chemical Sciences, University of Catania, Viale A. Doria 6, 95125 Catania, Italy; ncardullo@unict.it (N.C.); v.muccilli@unict.it (V.M.); 2 Department of Biological, Chemical, and Pharmaceutical Science and Technology (STEBICEF), University of Palermo, Via Archirafi 32, 90123 Palermo, Italy; vita.distefano@unipa.it; 3 Department of Health Sciences, University Magna Græcia of Catanzaro, Viale Europa, Germaneto, 88100 Catanzaro, Italy; s.bonacci@unicz.it; 4 National Council of Research, Institute of BioEconomy (CNR-IBE), Via Paolo Gaifami 18, 95126 Catania, Italy; lucia.sollima@cnr.it

**Keywords:** inulin, durum wheat, Sicilian landraces, spaghetti, quality, aminoacids, glycemic index, α amylase

## Abstract

Inulin is considered a dietary fiber and represents a noteworthy ingredient for food biofortification due to its health effects and its neutral taste. The aim of the work was the evaluation of the quality of pasta produced using whole-meal flours of two ancient Sicilian landraces (Senatore Cappelli-CAP and Timilia—TIM) fortified with two types of inulin (long-chain topinambur inulin IT and low-chain chicory inulin IC), at two different levels of substitution (2 and 4%) to evaluate its possible effect on α-amylase inhibition. The color indices L* and a* were mainly influenced by cultivars, while IT improved the sensory attributes, mainly the elasticity sensation, and influenced less the other sensory attributes: adhesiveness, color, odor, taste, and Over Quality Score for both landraces. The cooking quality was linked mainly to the landrace used, due to the very different gluten matrix of CAP and TIM. IC and IT showed promising α-Amy inhibitory activity with comparable IC_50_ values of 0.45 ± 0.04 and 0.50 ± 0.06 mg/mL. The enrichment of spaghetti with inulin with an inhibitory effect on α-amylase determined the hypoglycemic properties of pasta, thus lowering the corresponding IC_50_ value.

## 1. Introduction

Inulin is a type of fructan constituted by fructose units that feature mostly or exclusively the β (2 → 1) fructosyl-fructose linkage. It is essentially a linear polymer but can branch with β (2 → 6) linkages at a low degree of polymerization (DP). Inulin might also be useful in food promoting health effects [1,2]. In Dicots, the polymer is stored in taproot, bulbs, and capitula and its polymerization degree is related to the species and phenological stage of the plant [3,4,5,6,7,8,9].

Inulin is considered a dietary fiber [10], analogous to carbohydrates, that play a key role in human health for their special features: resistance to digestion and absorption in the human small intestine with complete or partial fermentation by the microflora in the colon, and the production and absorption of fermentation end products like short-chain fatty acid and lactate [11,12]. Inulin represents a noteworthy ingredient for food biofortification due to the health effects and its neutral taste. It does not create an off-flavor or aftertaste and can be used as a substitute for butter or margarine in bakery products [13] and dairy products [14]. 

Pancreatic α-amylase is a hydrolytic enzyme involved in the metabolism of starch. α-amylase catalyzes the hydrolysis of α-(1,4)-D-glycosidic linkages present in glycogen, amylopectin starch, amylose, dextrin, and maltose into other polymers of glucose [15]. Excessive conversion can increase the blood sugar level, and due to overactivation of α-amylase and deficiency of insulin, hyperglycemia condition can occur in some cases causing obesity, type II diabetes, and problems such as general overweight condition [15]. Various enzymatic inhibitors based on carbohydrate-related structures, such as acarbose, miglitol, and voglibose, effectively target this enzyme. However, there are some side effects such as diarrhea, flatulence, bloating, and abdominal discomfort, especially when administered in association with a high-carbohydrate diet, but these are reversible with discontinuation [16]. The side effects associated with the assumption of these drugs’ intake have led to the search for substitution therapies, based on natural or biocompatible molecules such as α-amylase inhibitors, which are efficient in contrasting hyperglycemia [17]. 

In contrast with starch, inulin and oligo-fructans are soluble and fermentable dietary fiber. As it is constituted of β-(2,1) bonds, inulin is resistant to hydrolysis by pancreatic α-amylase and reaches the large intestine completely unabsorbed [18]. Inulin is responsible for only 25–35% of energy compared to digestible carbohydrates, and thus it may be considered an appropriate ingredient for producing low caloric foods for people with diabetes to manage blood sugar levels [19]. Pasta is a staple food eaten daily or weekly in quantities constituting a dominant moiety of the diet in many countries, and it is regularly eaten in quantities that constitute a dominant portion of the diet worldwide, with 14.5 million tons produced in 2019 [20]. Pasta is favored by consumers for its versatility; ease of transportation, handling, cooking, and storage properties; availability in numerous shapes and sizes; high digestibility; good nutritional qualities; and relatively low cost. Therefore, pasta can be used as a carrier of specific compounds, such as inulin. The addition of the polymer to the flours could modify negatively the structure of fortified pasta compromising its acceptability by the consumers and the nutritional aspects [21,22], influencing the starch protein matrix, allowing more rapid access to the cooking water, gelatinizing the starch without affecting its swelling [23,24]. Aravind, 2012 [25] found that two inulin types with differing DP and crystallinity have different levels of integration with the starch-gluten matrix during pasta preparation. 

In our previous studies [13,22,26,27] the effects of inulin with two different DP and of durum wheat cultivars on quality, chemical composition, and glycemic index (GI) of spaghetti showed that the sensory characteristics of pasta were affected by inulin molecular weight and its interactions with the gluten matrix during pasta formation and they were significantly modified by polymerization degree; the effects of the various DP of inulin on the technological properties of the spaghetti were moreover influenced by the choice of the durum wheat cultivars. The DP affected most of the sensorial attributes of pasta as overall quality score (OQS) and in general, the increase in inulin amount determined a decline of sensory quality even though the molecular weight of inulin played a key role in pasta acceptability. This effect may be directly ascribed to the probable entanglements created by high molecular weight inulin that generally improved pasta characteristics; as consequence, inulin with high DP allowed the creation of a final pasta which was very interesting from a nutritional point of view and acceptable in terms of sensory properties and cooking quality. Regarding the effect of adding inulin, both the presence and the different DP of the inulin, the interaction with the cultivar with inulin was significant. The development of pasta produced using ancient Sicilian whole grains and enriched with a high content of dietary fiber would be a good way to increase fiber intake and reduce the GI of pasta and the development of gluten sensitivity. Considering the results on GI obtained using long-chain inulin and Timilia as durum wheat cultivar, in this work both the quality of pasta produced using whole-meal flours of two ancient Sicilian landraces fortified with two types of inulin with different degrees of polymerization at different levels of substitution (2 and 4%) and the possible effect on α-amylase inhibition have been evaluated. 

## 2. Results

### 2.1. Color, Sensory Attributes, and Cooking Quality

The organoleptic characteristic of inulin enriched pasta, as well as the cultivar of durum wheat used, could strongly influence this product, affecting consumer acceptability. The color indices of cooked spaghetti and the ANOVA analyses are reported in Table 1 and Table 2. The color indices L* and a* were mainly influenced by cultivars with the percentage of the total variation of 83.1% (L*) and 74% (a*). The percentage of substitution of inulin influenced the b* index, with 40% of the total variation, while the “Cultivar” influenced this parameter by 36.3%.

TIM presented a more brilliant color (L* = 66.1) while the yellow index b* was higher in CAP (L* = 16.4). The types of inulin did not influence the yellow index (b* = 15.4). 

The sensory attributes of cooked spaghetti samples were determined and shown in Table 3, as a percentage of variation vs. their relative CTRL. When averaged for used cultivars, both the CTRLs had all the scores for all the parameters higher than 5, the threshold of acceptability.

All the parameters resulted influenced by the cultivar and type of inulin. In particular, long-chain inulin improved mainly the elasticity sensation and influenced less the other sensory attributes: adhesiveness, color, odor, taste, and OQS.

The ANOVA for the technological properties of the samples is shown in Table 4. The OCT was influenced by the cultivar and by the percentage of inulin substitution in equal measure (42%); in general, the OCT decreased with the increase in the percentage of substitution, passing from 10.8 min (0%) to 8.7 min (4%). The cv TIM had a shorter cooking time than CAP, this type of semolina is most used to produce pasta, due to the tenacity of its gluten (Table 5).

Regarding water absorption, there were changes in these parameters between CTRLs and inulin-enriched samples. Specifically, TIM had the WA value of 106% vs. 152% of CAP. The cultivar influenced 85% of the total variation this parameter. Significant interactions “C X I” and “C X S” were observed. The swelling index resulted mainly influenced by the percentage of inulin substitution (51% of total variation) and it decreased with the increase of inulin. Cooking loss is influenced mainly by cultivar and is probably due to the competitive activity of inulin with the starch for water during pasta formation. The cooking loss in spaghetti samples was related mainly to the used cultivars (28.3% of the total variation) and partially to the percentage of inulin added. TIM attained the highest loss (5.41%), due mainly to its gluten matrix. 

### 2.2. Amminoacid Characterization

Aminoacids are essential nutritional components of a diet. They are found in food in the form of free aminoacids but above all as building blocks of proteins. Some operations are necessary for amino acid analysis in food, including hydrolysis of proteins, separation of individual aminoacids, and their quantification. The most established analytical techniques use HPLC (ion exchange or reverse-phase) and GC-MS methods, which require the derivatization of the sample [28]. Various reagents are used to derivatize the hydrolyzate before separation. Derivatization with 9-fluorenylmethyl chloroformate (FMOC-Cl) was used as pre-column method. The derivatives were separated on reversed-phase HPLC columns and the quantification was based on their fluorescent properties [29,30,31].

ANOVA (Table 6) showed that the landraces used and the percentage of inulin substitution affected the contents of L-alanine, L-leucine, and L-methionine, while L-phenylalanine was affected only by the percentage of substitution. All the other detected aminoacids were not affected by the studied factors. Moreover, interaction among the three studied factors was not found to be significant.

In Table 7 it is possible to observe that in CAP the aminoacid present in the highest concentration was L-alanine (1.99 g/100 g of spaghetti). L-histidine follows in order of concentration (1.83/100 g of spaghetti). L-phenylalanine (0.07 g/100 g of spaghetti g) was the aminoacid present in a lower concentration. Spaghetti obtained from durum wheat flour of TIM contains the aminoacid L-histidine in higher concentration (1.78 g/100 g of spaghetti). L-cystine and L-alanine follow in order of concentration (1.48 and 1.32 g/100 g of spaghetti respectively). As CAP the L-phenylalanine (0.07 g/100 g of spaghetti) was the aminoacid present in lower concentrations.

Except for L-cystine, L-lysine, and L-histidine, a decrease in the concentration of all detected aminoacids was observed, justified by the decrease in durum wheat flour replaced by oligosaccharide, but it is worthwhile to note that a good level of L-lysine was observed in both the durum wheat landraces and it was maintained when IC or IT inulins were added.

### 2.3. Inhibitory Effects of the Inulin Enriched Spaghetti on the Activity of α-Amylase

Commercial inulin from chicory (IC), inulin extracted from topinambur tubers (IT), spaghetti produced with cv Senatore Cappelli flour (CAP) and cv Timilia (TIM) used as control (CTRL), and the spaghetti obtained from the two whole-meal flours enriched with either 2% (CAP IC 2; CAP IT 2; TIM IC 2; TIM IT 2), and 4% (*w*/*w*; CAP IC 4; CAP IT 4; TIM IC 4; TIM IT 4) of the two types of inulin were evaluated. The inhibitory activity towards α-amylase from the porcine pancreas (α-Amy) was obtained using 2-chloro-4-nitrophenyl-α-D-maltoside (CNPG3) as substrate. The results are reported in Table 8 as the concentration (mg/mL) inhibiting the 50% of enzyme activity (IC_50_); the lower the value of IC_50_, the higher the inhibition is observed. The antidiabetic drug acarbose has been employed as a positive control.

With this assay, the inhibition of the enzyme is evaluated based on the concentration of the chromophore group that originates from the hydrolysis of the substrate CNPG3. 

During digestion, the digestible starch in pasta is degraded to maltose by α-amylase with a consequent postprandial blood glucose level rapidly increasing. When a pasta sample is analyzed for this inhibition assay, the starch content once placed in water is hydrolyzed by the enzyme in the place of the employed substrate. For the above reason, the inhibition assay was performed with CNPG3 as a substrate [32] employing the procedure described in the experimental section rather than the commonly applied method for determining the α-amylase inhibitory activity using starch as substrate [33,34].

Consequently, the pasta samples produced with cv Senatore Cappelli (CAP-CTRL) and cv Timilia (TIM-CTRL) flour used as control showed IC_50_ values of 3.94 ± 0.94 mg/mL and 2.92 ± 1.07 mg/mL as a result of their starch content. As reported in Table 8, IC and IT showed promising α-Amy inhibitory activity with comparable IC_50_ values of 0.45 ± 0.04 and 0.50 ± 0.06 mg/mL.

Thus, the enrichment of spaghetti with functional components with an inhibitory effect on α-amylase may determine the hypoglycemic properties of pasta, thus lowering the corresponding IC_50_ value.

Indeed, the enzymatic activity significantly decreased with the increasing concentration of the inhibitor. When the flour was enriched with IC (2 and 4%), the IC_50_ values were 3.86 ± 0.20 and 2.70 ± 0.52 mg/mL for CAP spaghetti, corresponding to an increase of the inhibitory activity from 2 to 31% with respect to CAP-CTRL. CAP-enriched with IT inulin led to an increase of the inhibitory activity from 37 to 43%. On the contrary, when TIM flour was enriched with 2% and 4% IC, the resulting spaghetti showed IC_50_ values of 2.11 ± 0.60 and 1.68 ± 0.71 mg/mL, respectively, corresponding to a higher increase in the inhibitory activity from 27 to 42% and IC_50_ values of 2.21 ± 0.78 and 1.97 ± 0.20 mg/mL, corresponding to an increase in the inhibitory activity from 24 to 32%. The good tenacity of the gluten of the CAP whole-meal flours better retains the long-chain inulin and this reflects the lower inhibitory activity of α-Amylase both at 2 and 4% vs. commercial inulin. TIM-CTRL is used mainly for bread production and it is characterized by a lower gluten tenacity and lower GI than other Sicilian landraces had the IC_50_ value reduced vs CAP-CTRL and interact better with IC.

The present results show how pasta enriched with inulin could positively slow down carbohydrates’ digestion/absorption process.

**Figure 1:**
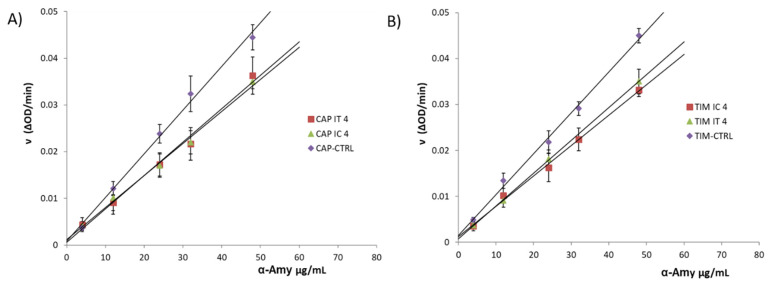
The reversibility effect of (**A**) CAP-CTRL and CAP-IC4 and CAP-IT4; (**B**) TM-CTRL and TIM-IC2 and TIM-IT2 on the α-Amylase activity.

Kinetic analyses were conducted to investigate the reversibility of enzymatic inhibition of the spaghetti with a higher amount of IC and IT compared to the corresponding control samples (Figure 1). The slopes of curves related to the CAP and TIM spaghetti enriched with IC and IT decreased than that of the respective control (CAP-CTRL and TIM-CTRL). Likewise, the straight lines of each group intersected the y-axis at 0, suggesting that the inhibiting process is reversible [35].

**Figure 2:**
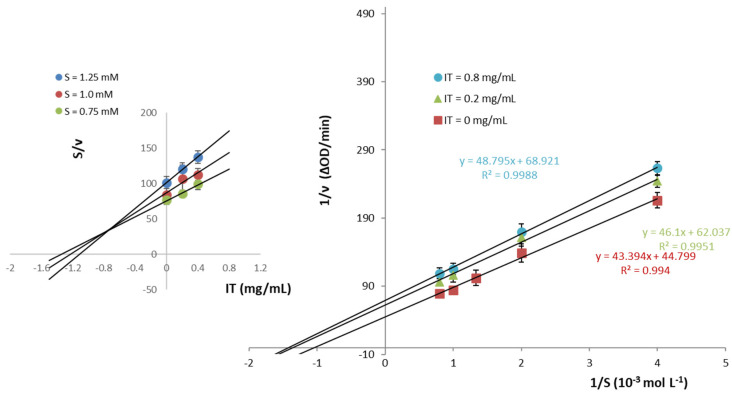
Lineweaver-Burk (L-B) of α-Amylase in the presence of inulin from topinambur (IT). The inset depicts the Cornish-Dowden plot to determine the *K’*_i_ constant.

**Figure 3:**
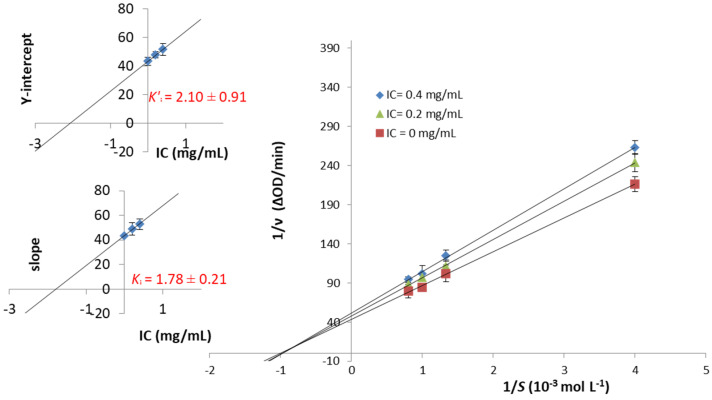
Lineweaver-Burk (L-B) of α-Amylase in presence of commercial inulin (IC). The insets depict the secondary plots to determine the kinetic constants.

Based on the above results the mechanism of α-Amylase inhibitory activity employing UV spectroscopy in the presence of inulin from topinambur (IT) was inspected, plotting the reciprocal of initial velocity (*ν*) versus the reciprocal of substrate concentration (S) at different concentrations of the inhibitor (Figure 2). Lineweaver–Burk (L-B) double reciprocal plot was generated from the acquired data to determine the inhibition type. L-B plot was also obtained for IC for comparison (Figure 3).

Linear relationships between 1/v and 1/S were observed in the plots. L-B plot obtained for IT indicated an un-competitive inhibition behavior at increasing concentration of IT (from 0 to 0.8 mg/mL) yielding parallel straight lines (Figure 2). Furthermore, the un-competitive inhibition was corroborated by Cornish-Dowden plot, where the data reported as S/v vs. IT concentration gave straight lines crossing in the second quadrant [35]. From the secondary plot, a dissociation constant *Kʹ*_i_ of 0.76 mg/mL related to the interaction of inhibitor (I) with the enzyme-substrate complex (IES) was determined.

Conversely, both the slopes (*K*_m_/v_max_) and Y-axis intercepts (1/v_max_) of curves of IC increased when the IC concentration increased from 0.0 to 0.4 mg/mL (Figure 3). All the curves intersected at the second quadrant, suggesting IC inhibits α-Amylase through a mixed-type mode that is an intermediate mechanism between non-competitive and competitive inhibition, agreeing with a previous report [35]. The secondary plots of the L-B graphic gave the *Kʹ_i_* and *K_i_* values, thus confirming the above-described mechanism.

## 3. Discussion

The landraces and the ancient varieties of durum wheat have gained, especially in Sicily, new attention, presumably thanks to the increased public awareness of environmental issues and the increased consumers’ demand for genuine and traditional foods. The pasta or bread obtained with these flours are generally perceived by consumers to be safer than those obtained from the modern varieties [36]. Furthermore, these products possess organoleptic, nutritional, and health-promoting properties, due to the presence of volatile organic compounds [37] and the higher total phenolic acid compounds and antioxidant activity [38,39]. However, the comparison of nutritional and nutraceutical value for ancient and modern durum wheat genotypes is still controversial, indicating a need for further research [36]. It is commonly stated that pasta made with ancient durum wheat cultivar possesses hypoglycemic properties, but Pandolfo et al. [40] compared the GI among pasta produced with commercial variety or with Sicilian landraces. The results do not support this popular idea of a reduced glycemic response elicited by wheat landrace pasta, attributed to the gluten net, the presence of “farinette”, and the operating system. Adding functional ingredients to these types of flours with beneficial effects can lead to the development of functional pasta, with hypoglycemic effects. In this work, two old varieties “Timilia”, an indigenous landrace from Sicily, and Senatore Cappelli, an Italian selection from the North-African landrace Jean Rhetifah [39], were used to obtain new products with hypoglycemic effects. In particular, commercial inulin from chicory (IC), inulin extracted from topinambur tubers (IT), spaghetti produced with cv Senatore Cappelli flour (CAP) and cv Timilia (TIM) used as control (CTRL), and the spaghetti obtained from the two whole-meal flours enriched with either 2% (CAP IC 2; CAP IT 2; TIM IC 2; TIM IT 2) or 4% (*w*/*w*; CAP IC 4; CAP IT 4; TIM IC 4; TIM IT 4) of the two types of inulin were evaluated for their sensory properties, for cooking quality, for aminoacids characterization, and α-Amy inhibition. 

The organoleptic characteristic of inulin enriched pasta, as well as the cultivar of durum wheat, used, influenced this product affecting consumer acceptability. In contrast to the previous study [22,26,27], where the addition of long-chain inulin to semolina was purified from Cynara cardunculus roots, the percentage of substitution of inulin influenced the b* index in both the durum wheat landraces used. All the produced pasta samples showed an OQS > 5 and IT inulin improved some sensory traits as already reported for long-chain inulin in previous studies, evaluating long-chain inulin from cardoon added to different cultivars of durum wheat semolina. The authors showed that long DP inulin addition improved or maintained elasticity, firmness, bulkiness, adhesiveness, color, odor, and the OQS scores. TIM maintained the OQS scores after inulin addition. Finally, there were no effects of inulin addition on odor and taste scores [10,25]. The type of inulin did not affect the swelling index, confirming the data reported Garbetta et al. [26] and partially agreeing with the results obtained by Aravind et al. [25] showing that samples fortified with high molecular weight inulin (high polymerization degree) recorded no changes in the swelling index, pasta water absorption, and cooking loss at the amount ≤10% vs. control sample. The types of inulin influenced the cooking loss. CI samples showed the highest cooking loss values. Inulin at low DP could be leaching from the pasta during cooking, which would contribute to the cooking loss, as also reported by other research teams [10,22,25,41].

Aminoacid composition is an important feature in determining the nutritional value of durum wheat grain for human and animal diets. Despite the strong interest in ancient durum wheat cultivars, the chemical characterizations mainly concerned the determination of macromolecules, antioxidants, and VOCs. No existing studies have aimed at determining the essential aminoacids of these ancient durum wheat cultivars, especially for lysine, one of the essential aminoacids that cannot be synthesized by humans and must therefore be obtained entirely from dietary sources. Thus, lysine content constitutes an important feature for defining the nutritive value of flour obtained from cereals. The results of quali-quantitative determination of aminoacids in spaghetti samples showed that the used method was capable of separating aminoacids derivatives. Seven aminoacids including L-alanine, L-leucine L-methionine L-phenylalanine L-cystine, L-lysine, and L-histidine were identified and quantified in spaghetti samples using external standards calibration curves (Table S1). The data obtained in this work for aminoacids quantification showed that the addition of the polymer to pasta, regardless of the polymerization degree, slightly declined the percent of aminoacids as the concentration of inulin increased. The data agree with the decline in total protein content recorded in previous work [22], even if the lysine content was maintained. Lysine generally represents 2% of total protein [42] and the data obtained agree with the dataset of CREA, food code 000805 [43]. 

The physical structure of the gluten matrix, formed by durum wheat starch and wheat proteins, is the main intrinsic factor supposed to explain the lower glycemic response of 100% refined wheat pasta products, even if the overall concept of the low GI of durum wheat pasta should be contextualized with the raw materials (common or durum wheat, refined or whole wheat), their origin, and the technological process used to produce it, rather than with the experimental conditions (i.e., sample size, characteristics and dietary patterns of the enrolled subjects, and inter-day variability) applied throughout the study [40]. The results of the proteomic profile and qualitative comparison at the molecular level of metabolic and chloroform-methanol (CM)-like protein fractions of the old Sicilian landraces Russello and Timilia Reste Bianche and the modern cultivar Simeto reveal that the metabolic and CM-like protein fractions of old and modern genotypes are remarkably similar, except for the α-Amylase/trypsin inhibitor CM1 and CMd only identified in Timilia [36]. In our study, the potential hypoglycemic effect of pasta produced with ancient durum wheat flours and inulin was evaluated by the α-Amy inhibition; the enzyme activity resulted linked to the landraces used, and substituting inulin to the flours, allowed us to obtain high percentages of the α-Amy inhibition, also confirming the data previously obtained on GI for TIM spaghetti added by cardoon long-chain inulin [26] and for the starch digestibility of CAP spaghetti supplemented with high DP inulin, that turned out to be smaller than that of samples with low DP inulin [22]. In particular, the in vitro results of α-Amy inhibition have highlighted a reduction of enzyme activity in a dose-dependent manner with the inulin presence in pasta samples. Previous findings pointed out that soluble fibers, such as inulin-type fructans, can have a serum glucose-lowering effect [44]. Our study supports these data as we have observed a lowering of α-Amy IC_50_ values with an increasing % of inulin included in pasta. Furthermore, kinetic studies corroborated the glucose-lowering effect of fructan-based fibers. The different mechanisms of inhibition observed for commercial inulin (IC) and that obtained from topinambur (IT) may be explained by the different composition and DP values of the two inulins. Moreover, the kinetics data plotted in Figure 1 clearly show a reduction in the reaction rate between α-Amy and the substrate (CNPG3) when pasta is prepared with flour (both TIM and CAP) enriched with 4% inulin.

Because non-digestible oligosaccharides of long-chain length are typically less (or more slowly) biodegradable than compounds of shorter chain length inulin, fermentation will take a longer time than oligofructose fermentation, which results in more residual carbohydrate breakdown in the distal colon compartments. Due to the good results obtained in terms of sensory attributes, aminoacid content and α-Amy inhibition future research will be directed towards increasing the amount of long-chain inulin in flours, evaluating also the effects on gut microbiota.

## 4. Materials and Methods

### 4.1. Raw Material

Inulin was extracted and characterized from tubers of *Helianthus tuberosus*, population “Pennisi”, cropped in the experimental field of Cassibile on the coastal plain, south of Syracuse (37°03′ N, 15°18′ E, 15 m a.s.l.) during the year 2018. Tubers were collected at marketable maturity.

In the laboratory was performed the extraction, purification, and characterization of inulin to calculate the DP and for spaghetti preparation. The moisture content of a representative sample of tubers was measured after drying the plant material to a constant weight in a thermo-ventilated drying oven at 105 °C. Tubers were washed in cold tap water, scraped, and ground to a fine powder. The processes of extraction and purification are described in Padalino et al. [22]. Briefly, 100 g of the original homogenate was diluted with water and put in a boiling water bath for 30 min. After cooling to room temperature, the extract was filtered and centrifuged at 3000× *g* for 5 min. The inulin extracted was precipitated at 0 °C overnight. The supernatants were removed and inulin was washed with distilled water and precipitated at 0 °C overnight. The washing process was repeated until inulin was white. The color was determined by colorimeter Minolta CR 400, and L* values above 85 were accepted for purification. Furthermore, inulin was lyophilized in Petri dishes and used for pasta production. The moisture content in lyophilized inulin was determined in a thermo-ventilated oven at 105 °C, resulting in less than 0.5 g/100 g of fresh weight. Inulin characterization and quantification were performed following the method of Sillitti et al. [27]. The maximum degree of polymerization recorded was, in this plant phenological phase, 80 fructose units, with a mean DP of 50 fructose units. Commercial inulin that is manufactured by ORAFTI (Tienen, Belgium) as Raftilose^®^ Synergy 1 is an oligofructose enriched inulin. This is a 1/1 mixture of long-chain and short chain fractions of inulin extracted from chicory roots (*Cichorium intybus*). Inulin is made by a set of linear chains of fructose molecules, with a DP ranging between 3 and 65. It can be fractionated into a slowly fermentable long-chain fraction (DP ranging from 10 to 65, average 25) or in a rapidly fermentable fraction made of oligofructose (DP ranging from 3 to 8, average 4). Synergy1 is a mixture of both fractions and has a higher number of long chains relative to the native product. 

### 4.2. Spaghetti Preparation

Spaghetti was produced with 2 durum wheat semi whole-meal flour: cv Senatore Cappelli (CAP) and cv Timilia (TIM). For each cv (see Table 9), pasta without inulin was used as control (CTRL), by using the operating conditions described by Melilli et al. [45]. Semi whole-meal flour of CAP was characterized by 2.1% (fat), 66.3% (carbohydrates), 12.7% (proteins), and 7.7% (fiber), while TIM was characterized by 1.8% (fat), 68.0% (carbohydrates), 12.0% (proteins), and 5.5% (fiber). The flours were mixed with water substituting either 2% or 4% (*w*/*w*) of the different types of inulin as powder (Table 9): inulin extracted from topinambur tubers (IT) and commercial inulin from chicory (IC) (Orafti^®^). To ensure the solubility of the inulin powder, they were previously dissolved in water. The dough was extruded into a spaghetti shape (30 cm in length × 1.70 mm).

### 4.3. Color

Color was measured in spaghetti after cooking. Pasta color data were collected using a Chroma Meter (Minolta CR-400, Milan, Lombardy, Italy). The spaghetti samples were arranged close together in rows. The colorimeter was calibrate using the manufacturer’s standard white plate (L* = 96.55; a* = −0.35; b* = −0.16) [45,46].

### 4.4. Sensory Analysis and Spaghetti Cooking Quality

Dry spaghetti samples were submitted to a panel of fifteen trained tasters (six men and nine women, aged between 28 and 45) to evaluate the sensory attributes. The panelists were selected based on their sensory skills (ability to accurately determine and communicate the sensory attributes such as appearance, odor, taste, and texture of a product). The panelists were also trained in sensory vocabulary and identification of particular attributes by evaluating durum wheat commercial spaghetti (ISO 11036, 7304). They were asked to indicate elasticity, firmness, bulkiness, adhesiveness, fibrous nature, color, odor, and taste for cooked spaghetti [26,28]. To this aim, a nine-point scale, where 1 corresponded to *extremely unpleasant*, 9 to *extremely pleasant*, and 5 to the *threshold acceptability*, was used to quantify each attribute [47]. Based on the above-mentioned attributes, panelists were also asked to score the overall quality (OQS) of the product using the same scale.

The optimal cooking time (OCT), the cooking loss, and the amount of solid substance lost into the cooking water were evaluated according to the AACC-approved method 66–50 (2000). The swelling index of cooked pasta was determined according to the procedure described by Cleary & Brennan (2006). The swelling index was expressed as follows:

(weight of cooked spaghetti) − (weight of spaghetti after drying)/(weight of spaghetti after drying).

The water absorption of drained pasta was also determined as follows:

(weight of cooked spaghetti) − (weight of raw pasta)/(weight of raw pasta)

Three measurements were performed for each analysis, and the mean values were calculated.

### 4.5. Aminoacid Determination

The use of HPLC-FLD in determining derivatized aminoacids was studied. Approximately 500 mg of spaghetti sample with 1 mL of HCl 6 M at 110 °C was transferred to a clean hydrolysis tube for 24 h. After cooling, the contents were transferred to a 2 mL Eppendorf reaction vessel and evaporated to dryness at 60 °C. The pellet was dissolved in 1 mL of high-purity water and solution filtered with 0.45 µm PTFE syringe filters. Aminoacids mixture was derivatized at room temperature using a precolumn procedure using FMOC-Cl. The mixture was quickly shaken and the reaction was allowed to proceed for 10 min at 70 °C.

Excess FMOC-Cl was destroyed by the addition of 50 µL of eptilamine solution (3 mL eptilamine, 15 mL ACN e 175 mL HCl 0.1 M, pH 9) and after vortexed for 3 min.

Then, 80 µL of the solution were withdrawn and 320 µL of ACN and 600 µL of hexane were added. Finally, 20 µL of the final solution containing the derivatized aminoacids was injected into the HPLC-FLD instrument. HPLC separation was carried out in an Agilent 1100 liquid chromatographic system equipped with a Fluorescence detector (model G1321A), controlled by Chemstation software. A selected Discovery HS C18 analytical column (3.5 μm particle size, 150 mm × 4.6 mm i.d.) (Supelco, Bellefonte, PA, USA) fitted with guard column was chosen. The column operated at 40 °C with a flow rate of 1.0 mL/min using water containing 0.1% formic acid as eluent A and ACN as eluent B. The gradient elution program was: 0–10 min 3% B; 3–17 min linear increase to 10% B; 17–47 min linear increase to 50% B; 47–57 min linear increase to 100% B, 57–60 min hold 100% B; 60–63 min returning to the initial conditions and being equilibrated. Each derivative eluted from the column is monitored by a fluorometric detector (FLD) set at an excitation wavelength of 254 nm and an emission wavelength of 630 nm.

Peak identification was based on the comparison between the retention time of the standards of the aminoacids and those in spaghetti samples and was confirmed by a fortification technique (spiking). Quantitation was based on the external standard method using calibration curves fitted by linear regression analysis. The calibration solution was based on a mix of 17 aminoacids, NIST standard reference material (NIST 2389a): aminoacids (2.50 ± 0.07 mM L-alanine, 2.51 ± 0.07 mM/L L-arginine, 2.50 ± 0.08  mM/L L-aspartic acid, 1.23 ± 0.06  mM/L L-cystine, 2.50 ± 0.08  mM/L L-glutamic acid, 2.52 ± 0.07  mM/L glycine, 2.52 ± 0.07 L-histidine, 2.44 ± 0.11  mM/L L-isoleucine, 2.44 ± 0.11  mM/L L-leucine, 2.41 ± 0.17  mM/L L-lysine, 2.51 ± 0.07  mM/L L-methionine, 2.55 ± 0.09  mM/L L-phenylalanine, 2.46 ± 0.11  mM/L L-proline, 2.44 ± 0.11  mM/L L-serine, 2.49 ± 0.07  mM/L L-threonine, 2.54 ± 0.08  mM/L L-tyrosine, and 2.51 ± 0.10  mM/L L-valine) in 0.1 N HCl. L-Tryptophan, L-asparagine, and L-glutamine have been taken from the L-aminoacid set (Sigma-Aldrich LAA21). To verify the linearity of the response of different aminoacids derivatives, standard solutions of a set of 17 aminoacids in the concentrations from 0.1 μM/L to 1 μM/L and from 0.25 mM/L to 1.87 mM/L for tryptophan, asparagine, and glutamine, were prepared and analyzed in triplicate. Linearity was evaluated by the determination coefficient of the least square regression (R^2^). The coefficient of regression exceeded 0.934 for all standard calibration curves. The results indicated that aminoacids derivatives showed good linearity under the proposed conditions. 

### 4.6. In Vitro Pancreatic α-Amylase Inhibition and Kinetic Assay

The inhibition of the porcine pancreatic α-Amylase (EC3.2.1.1, Type VI-B; α-Amy) was achieved according to the method described by Proença et al. [32] with some modifications. 

The assay is based on the evaluation of the hydrolysis of the substrate 2-chloro-4-nitrophenyl-a-D-maltotrioside (CNPG3) into 2-chloro-nitrophenol (CNP), 2-chloro-4-nitrophenyl-α-D-maltoside (CNPG2), maltotriose and glucose. The initial amount of released CNP is proportional to the concentration of α-Amy present. The assay was performed in a 96-well plate (final volume 200 µL) with the enzyme (0.08 mg/mL) dissolved in 100 mM phosphate buffer (with 0.03% NaN_3_; pH 6.8). Samples were dissolved in hot water (IC and IT = 2 mg/mL; other samples 20 mg/mL) under magnetic stirring for 1 h and 30 min and added (10, 20, 40, 60 μL) to the enzyme (10 µL). The plate was incubated for 10 min at 37 °C. The optical density (OD) was monitored at 405 nm with the Synergy H1 microplate reader (BioTek, Bad Friedrichshall, Germany) for 30 min, at 37 °C after the addition of CNPG3 (10 mM, 20 µL), dissolved in phosphate buffer. Acarbose (0.05 mg/mL) was used as a positive control. The inhibition percentage was obtained with the following equation:

(1) $$inhibition\;\%=\frac{\left(OD_{control}-OD_{sample}\right)}{OD_{control}\;}\times 100$$

where *OD_control_* and *OD_sample_* are the optical density measured for the mixture enzyme/substrate and the mixtures enzyme/inhibitor/substrate, respectively. Optical density values obtained at 30 min were employed for the calculation by regression analysis of the concentration required to inhibit the 50% activity of the enzyme (IC_50_). The results were expressed as mean ± standard error of the mean.

Kinetic assays were used to investigate the reversibility of α-Amylase inhibitory activity in presence of pasta and pasta with inulin samples as previously reported [19]. The concentrations of the samples (1 mg/mL) and the substrate (1 mM) were kept constant, while the enzyme concentration ranged between 4 and 60 µg/mL. 

A similar procedure was adopted to evaluate the mode of inhibition and the inhibitory constants for IC and IT. Specifically, mixtures containing α-Amylase (10 μL of a 0.1 mg/mL solution), IC or IT (0.2, 0.4, and 0.8 mg/mL), and CNPG3 at different concentrations (0, 0.25, 0.5, 0.75, 1.0, and 1.25 mM) were incubated at 37 °C, and the optical density was read at 405 nm every 1 min for 30 min with the microplate reader. The initial velocity (*ν*) was determined as the slope of the OD changes during the linear course of the reaction. 

The kinetic Equation (2) is reported in a double reciprocal form (Equation (3)) to give the Lineweaver-Burk plots for competitive inhibitors.

(2) $$v=\frac{v_{max}S}{K_{m}\;\left(1+\frac{I}{K_{i}}\right)+S\;}$$

(3) $$\frac{1}{v}=\frac{K_{m}\;\left(1+\frac{I}{K_{i}}\right)}{v_{max}}\times \frac{1}{S}+\frac{1}{v_{max}\;}$$

Equation (4) can be reported in a double reciprocal form (Equation (5)) to obtain the Lineweaver-Burk plots for mixed-type inhibitors:

(4) $$v=\frac{v_{max}S}{K_{m}\;\left(1+\frac{I}{K_{i}}\right)+S\;\left(1+\frac{I}{{K^{\prime }}_{i}}\right)}\;$$

(5) $$\frac{1}{v}=\frac{K_{m}\;\left(1+\frac{I}{K_{i}}\right)}{v_{max}}\times \frac{1}{S}+\frac{\left(1+\frac{I}{{K^{\prime }}_{i}}\right)}{v_{max}\;}$$

Equation (6) can be reported in a double reciprocal form (Equation (7)) to obtain the Lineweaver-Burk plots for un-competitive inhibitors

(6) $$v=\frac{v_{max}^{\prime }S}{S+{K^{\prime }}_{m}\;}$$

(7) $$\frac{1}{v}=\frac{{K^{\prime }}_{m}\;}{v_{max}^{\prime }}\times \frac{1}{S}+\frac{1}{\begin{array}{c} v_{max} \end{array}}$$

where *ν* and *ν_max_* are the enzyme reaction initial rate and the maximal velocity, respectively, in the absence and presence of inhibitors; *ν*′*_max_* is the value of *ν_max_* in the presence of an initial concentration of un-competitive inhibitor. *K_m_*, *K_i_*, and *K*′*_i_* are the Michaelis-Menten, the competitive, and non-competitive inhibition constants, respectively; *K*′*_m_* is the apparent value for *K_m_*; S and I are substrate (CNPG3) and inhibitor concentrations.

*K_i_*, and *K*′*_i_* values can be obtained with the secondary plots obtained from Equations (3), (5) and (7). 

### 4.7. Statistical Analysis 

Data were submitted to Bartlett’s test for the homogeneity of variance and then analyzed using analysis of variance (ANOVA). Means were statistically separated based on Tukey’s test or Duncan’s test when the ‘F’ test of ANOVA for treatment was significant at least at the 0.05 probability (CoHort Software, CoStat version 6.451). 

## Figures and Tables

**Table 1:** Sum of square (SS) and percentage of variation (%) of spaghetti samples color indices (L*, a*, and b*) in relation to the studied factors.

Source of Variation	L*			a*			b*		
	SS	%	p	SS	%	p	SS	%	p
Cultivar (C)	423	83.1	***	23.6	74.0	***	23.6	36.3	***
Inulin Type (I)	0.01	0	ns	0.02	0.1	ns	0.23	0.4	ns
Inulin substitution (S)	0.79	0.20	ns	1.22	3.8	***	25.98	40.0	***
Interaction									
C X I	2.05	0.40	ns	0.23	0.7	**	0.06	0.1	ns
C X S	80.6	15.8	**	6.28	19.7	***	0.51	0.8	ns
I X S	1.63	0.30	ns	0.38	1.2	**	7.2	11.1	**
C X I X S	1.10	0.20	ns	0.16	0.5	*	7.4	11.4	**

L*: lightness, a*: redness, and b*: yellowness; ***, **, and * indicate significant at *p* < 0.001, *p* < 0.01, and *p* < 0.05, respectively, and “ns” indicates not significant.

**Table 2:** Color indices of cooked spaghetti (mean ± SD, *n* = 3) as affected by cultivar, inulin-type, and percentage of substitution.

Sample	L*	a*	b*
**Cultivar**			
CAP	57.7 ± 2.08 b**	4.51 ± 0.16 a	16.4 ± 0.59 a
TIM	66.1 ± 2.36 a	2.52 ± 0.09 b	14.4 ± 0.51 b
**Inulin**			
IC	61.9 ± 2.23 a	3.53 ± 0.12 a	15.5 ± 0.52 a
IT	61.8 ± 2.23 a	3.51 ± 0.12 a	15.3 ± 0.53 a
**Percentage of substitution**			
0%	62.1 ± 2.24 a	3.83 ± 0.14 a	16.7 ± 0.60 a
2%	62.0 ± 2.23 a	3.40 ± 0.12 b	15.4 ± 0.55 b
4%	61.7 ± 2.21 b	3.31 ± 0.11 c	14.1 ± 0.21 c

** Different letters in each column indicate statistical differences at *p* < 0.05 (Duncan’s test). L*: lightness, a*: redness, and b*: yellowness; IC: Chicory Inulin; IT: Topinambur Inulin.

**Table 3:** Sensorial attributes (mean ± SD; *n* = 15) of all cooked spaghetti. Different letters in each column indicate statistical differences at *p* < 0.05 (Duncan’s test).

Sample	Elasticity	Firmness	Fibrous	Bulkiness	Adhesiveness	Color	Odor	Taste	OQS
**Absolute values**									
CAP-CTRL	6.3	6.4	6.7	6.3	6.1	7.1	7.3	7.3	6.4
TIM-CTRL	5.7	6.0	6.0	6.3	6.1	7.2	7.5	7.2	6.3
**Percentage of variation vs. their relative CTRL**									
CAP IC 2	−4.4 ± 0.13 d	−6.0 ± 0.17 f	−2.2 ± 0.06 c	−6.1 ± 0.18 c	−4.5 ± 0.13 d	−4.5 ± 0.01 d	−3.2 ± 0.09 e	−0.5 ± 0.01 b	−5.3 ± 0.15 b
CAP IC 4	−6.9 ± 0.20 e	−2.9 ± 0.08 d	−1.0 ± 0.03 b	−10.9 ± 0.32 e	−12.4 ± 0.36 f	−12.4 ± 0.05 f	−4.3 ± 0.13 f	−1 ± 0.03 c	−12.2 ± 0.036 d
CAP IT 2	2.8 ± 0.08 b	−2.1 ± 0.06 c	0.1 ± 0.01 a	−4.1 ± 0.12 a	−1.7 ± 0.05 b	−1.7 ± 0.02 b	4.4 ± 0.13 a	−0.8 ± 0.02 c	−2.8 ± 0.08 a
CAP IT 4	−9.3 ± 0.27 f	−1.3 ± 0.04 b	−5.8 ± 0.17 e	−16.8 ± 0.49 f	−12.7 ± 0.37 f	−12.7 ± 0.05 f	1.8 ± 0.05 c	2.5 ± 0.07 a	−2.9 ± 0.08 a
TIM IC 2	−2.7 ± 0.08 c	−0.6 ± 0.02 a	−4.7 ± 0.14 d	−9.5 ± 0.28 d	−4.3 ± 0.13 d	−4.3 ± 0.11 d	1.6 ± 0.05 cd	−3.2 ± 0.09 g	−10.4 ± 0.30 c
TIM IC 4	7.6 ± 0.22 a	−0.8 ± 0.02 ab	−4.5 ± 0.13 d	−5.0 ± 0.15 ab	−8.3 ± 0.24 e	−8.3 ± 0.09 e	2.3 ± 0.07 b	−2.1 ± 0.06 e	−3.4 ± 0.10 a
TIM IT 2	7.8 ± 0.23 a	−5.0 ± 0.15 e	−5.8 ± 0.17 e	−6.0 ± 0.17 bc	−0.3 ± 0.01 a	−0.3 ± 0.05 a	1.3 ± 0.04 d	−2.8 ± 0.08 f	−3.2 ± 0.09 a
TIM IT 4	7.1 ± 0.21 a	−9.5 ± 0.28 g	−22 ± 0.64 f	−4.7 ± 0.14 a	−2.8 ± 0.08 c	−2.8 ± 0.06 c	1.3 ± 0.04 d	−1.4 ± 0.04 d	−3.2 ± 0.09 a
**Cultivar**									
CAP	−4.5 ± 0.17 b	−3.1 ± 0.09 a	−2.2 ± 0.07 a	−9.5 ± 0.28 b	−7.8 ± 0.23 b	0.5 ± 0.03 a	−0.3 ± 0.01 b	0.1 ± 0.03 a	−5.8 ± 0.17 b
TIM	5.0 ± 0.18 a	−4.0 ± 0.12 b	−9.3 ± 0.27 b	−6.3 ± 0.18 a	−3.9 ± 0.11 a	−2.7 ± 0.08 b	1.6 ± 0.05 a	−2.3 ± 0.07 b	−5.0 ± 0.15 a
**Inulin**									
IC	−1.6 ± 0.16 b	−2.6 ± 0.07 a	−3.1 ± 0.09 a	−7.9 ± 0.23 a	−7.4 ± 0.21 b	−1.5 ± 0.07 b	−0.9 ± 0.08 b	−1.7 ± 0.05 b	−7.8 ± 0.23 b
IT	2.1 ± 0.20 a	−4.5 ± 0.13 b	−8.4 ± 0.25 b	−7.9 ± 0.23 a	−4.4 ± 0.13 a	−0.7 ± 0.05 a	2.2 ± 0.06 a	−0.6 ± 0.05 a	−3.0 ± 0.09 a
**Percentage of substitution**									
2%	0.87 ± 0.13 a	−3.4 ± 0.10 a	−3.1 ± 0.09 a	−6.4 ± 0.19 a	−2.7 ± 0.08 a	−1.7 ± 0.05 b	1.0 ± 0.08 a	−1.8 ± 0.05 b	−5.4 ± 0.16 a
4%	−0.38 ± 0.22 b	−3.6 ± 0.11 a	−8.3 ± 0.24 b	−9.3 ± 0.27 b	−9.1 ± 0.26 b	−0.4 ± 0.06 a	0.3 ± 0.07 b	−0.5 ± 0.05 a	−5.4 ± 0.16 a
**ANOVA**									
*Main effects*									
Cultivar (C)	***	***	***	***	***	***	***	***	**
Inulin Type (I)	***	***	***	ns	***	***	***	***	***
Inulin substitution (S)	***	ns	***	***	***	***	***	***	ns
*Interaction*									
C X I	***	***	***	***	***	***	***	***	***
C X S	***	***	***	***	***	***	***	ns	***
I X S	***	***	***	***	ns	*	***	***	ns
C X I X S	ns	**	***	***	***	***	*	***	***

***, **, and * indicate significant at *p* < 0.001, *p* < 0.01, and *p* < 0.05, respectively, and “ns” indicates not significant.

**Table 4:** Sum of square (SS) and percentage of variation (%) of spaghetti samples cooking quality in relation to the studied factors.

Source of Variation	OCT			Water Absorption			Swelling Index			Cooking Loss		
	SS	%	p	SS	%	p	SS	%	p	SS	%	p
Cultivar (C)	21.1	42	***	12,493	85	***	0.28	29.1	***	1.09	28.3	***
Inulin Type (I)	1.76	3.5	**	286	2.0	**	0.05	5.21	ns	0.2	5.19	*
Inulin substitution (S)	21.1	42	***	688	4.7	***	0.49	51.0	***	0.56	14.5	***
Interaction												
C X I	1.26	2.5	**	429	2.9	***	0.05	5.21	**	0.07	1.82	**
C X S	3.93	7.7	***	335	2.3	**	0.01	1.04	ns	0.79	20.5	ns
I X S	0.89	1.8	*	143	1.0	ns	0.04	4.17	ns	0.16	4.16	ns
C X I X S	0.77	1.5	ns	246	1.7	*	0.04	4.17	*	0.98	25.5	*

***, **, and * indicate significant at *p* < 0.001, *p* < 0.01, and *p* < 0.05, respectively, and “ns” indicates not significant. OCT: Optimal Cooking Time.

**Table 5:** Cooking quality of spaghetti (mean ± SD, *n* = 3).

	OCT	Water Absorption	Swelling Index	Cooking Loss
**Cultivar**				
CAP	10.5 ± 0.41 a*	152 ± 10.2 a	2.03 ± 0.12 b	4.98 ± 0.57 b
TIM	8.62 ± 0.18 b	106 ± 9.8 b	2.25 ± 0.14 a	5.41 ± 0.41 a
**Inulin**				
IC	9.3 ± 0.35 b	133 ± 10.2 a	2.19 ± 0.23 a	5.10 ± 0.56 a
IT	9.8 ± 0.34 a	126 ± 10.6 b	2.09 ± 0.05 b	5.30 ± 0.35 a
**Percentage of substitution**				
0%	10.8 ± 0.18 a	122 ± 9.3 b	2.33 ± 0.13 a	5.31 ± 0.56 a
2%	9.1 ± 0.53 b	132 ± 13.2 a	1.99 ± 0.23 b	4.98 ± 0.49 b
4%	8.7 ± 0.18 c	134 ± 7.6 a	2.10 ± 0.04 b	5.30 ± 0.42 a

* Different letters in each column indicate statistical differences at *p* < 0.05 (Duncan’s test).

**Table 6:** ANOVA analyses for each aminoacid as affected by cultivar, inulin-type, and percentage of substitution.

Source of Variation	L-Alanine	L-Leucine	L-Methionine	L-Phenylalanine	L-Cystine	L-Lysine	L-Histidine
**Main effects**							
Cultivar (C)	***	***	***	ns	ns	ns	ns
Inulin Type (I)	ns	ns	ns	ns	ns	ns	ns
Inulin substitution (S)	***	*	**	***	ns	ns	ns
**Interaction**							
C X I	ns	ns	ns	ns	ns	ns	ns
C X S	ns	ns	ns	ns	ns	ns	ns
I X S	ns	ns	ns	*	ns	ns	ns
C X I X S	ns	ns	ns	ns	ns	ns	ns

***, **, and * indicate significant at *p* < 0.001, *p* < 0.01, and *p* < 0.05, respectively, and “ns” indicates not significant.

**Table 7:** Aminoacid content (g/100 g of spaghetti-mean ± SD, *n* = 3) as affected by cultivar, inulin-type, and percentage of substitution.

Sample	L-Alanine	L-Leucine	L-Methionine	L-Phenylalanine	L-Cystine	L-Lysine	L-Histidine
**Cultivar**							
CAP	1.99 ± 0.264 a	1.42 ± 0.134 a*	0.24 ± 0.054 b	0.07 ± 0.073 a	1.53 ± 0.021 a	1.00 ± 0.033 a	1.83 ± 0.013 a
TIM	1.32 ± 0.096 b	1.13 ± 0.061 b	0.28 ± 0.050 a	0.07 ± 0.139 a	1.48 ± 0.046 a	0.99 ± 0.025 a	1.78 ± 0.031 a
**Inulin**							
IC	1.69 ± 0.120 a	1.29 ± 0.096 a	0.26 ± 0.076 a	0.07 ± 0.191 a	1.51 ± 0.017 a	1.00 ± 0.031 a	1.80 ± 0.006 a
IT	1.62 ± 0.265 a	1.26 ± 0.146 a	0.27 ± 0.040 a	0.07 ± 0.072 a	1.50 ± 0.052 a	0.99 ± 0.035 a	1.80 ± 0.042 a
**Percentage of substitution**							
0%	1.87 ± 0.130 a	1.34 ± 0.006 a	0.28 ± 0.029 a	0.08 ± 0.007 a	1.52 ± 0.031 a	1.02 ± 0.014 a	1.82 ± 0.014 a
2%	1.67 ± 0.159 b	1.27 ± 0.082 ab	0.26 ± 0.076 a	0.07 ± 0.051 b	1.50 ± 0.040 a	0.99 ± 0.020 a	1.80 ± 0.009 a
4%	1.43 ± 0.226 c	1.06 ± 0.160 b	0.22 ± 0.040 b	0.05 ± 0.212 c	1.32 ± 0.029 a	0.86 ± 0.046 a	1.58 ± 0.039 a

* Different letters in each column indicate statistical differences at *p* < 0.05 (Duncan’s test).

**Table 8:** The α-amylase inhibitory activity of spaghetti produced with cv Senatore Cappelli flour (CAP) and cv Timilia (TIM) and spaghetti enriched with inulin samples (IC and IT).

Sample	α-Amy IC_50_ ± SD ^1^
IC	0.45 ± 0.04 a*
IT	0.50 ± 0.06 a
CAP-CTRL	3.94 ± 0.94 b,c
CAP IC 2	3.86 ± 0.20 b
CAP IC 4	2.70 ± 0.52 b,c
CAP IT 2	2.49 ± 0.93 b,c
CAP IT 4	2.23 ± 0.84 c
TIM-CTRL	2.92 ± 1.07 b,c
TIM IC 2	2.11 ± 0.60 c
TIM IC 4	1.68 ± 0.71 c
TIM IT 2	2.21 ± 0.78 c
TIM IT 4	1.97 ± 0.20 c
Acarbose	0.0033 ± 0.00015 d

^1^ Data are reported as mean ± SD (*n* = 3) of IC_50_, the concentration (mg/mL) required to inhibit the 50% of α-amylase activity. * Different letters (a–d) indicate statistical differences at *p* < 0.05 (Tukey’s test).

**Table 9:** Samples under study.

Durum Wheat Cv	Inulin Addition	Topinambur Inulin (IT)	Chicory Inulin (IC)
Senatore Cappelli	0	CAP-CTRL	
	2%	CAP IT 2	CAP IC 2
	4%	CAP IT 4	CAP IC 4
Timilia	0	TIM-CTRL	
	2%	TIM IT 2	TIM IC 2
	4%	TIM IT 4	TIM IC 4

## Data Availability

Not applicable.

## Supplementary Materials

The following supporting information can be downloaded at: https://www.mdpi.com/article/10.3390/molecules27082482/s1, Table S1: Retention time (min.), coefficient of determination (R^2^) and linear regression model of external standards used for calibration.

## Sample Availability

Samples of the compounds of the inulins are available from the authors.
