# Supplementary material for: Spaghetti Enriched with Inulin: Effect of Polymerization Degree on Quality Traits and α-Amylase Inhibition
Source: Molecules. 2022 Apr 12;27(8):2482. doi: 10.3390/molecules27082482 (PMC9026318; doi:10.3390/molecules27082482)
Supplement: Supplementary file 1 [file molecules-27-02482-s001.zip › molecules-1686672-supplementary.pdf]

Article

Spaghetti Enriched with Inulin: Effect of Polymerization Degree on Quality Traits and α-Amylase Inhibition

Nunzio Cardullo [1], Vera Muccilli [1], Vita Di Stefano [2], Sonia Bonacci[3] , Lucia Sollima [4] and Maria Grazia Melilli [4*]

[1] Dipartimento di Scienze Chimiche, Università degli Studi di Catania, V.le A. Doria 6, Catania 95125, Italy; ncardullo@unict.it (N.C.), v.muccilli@unict.it (V.M.)
[2] Department of Biological, Chemical, and Pharmaceutical Science and Technology (STEBICEF), University of Palermo, Palermo, Italy; vita.distefano@unipa.it (V.D.)
[3] Department of Health Sciences, University Magna Græcia of Catanzaro , Viale Europa, Germaneto, 88100 Catanzaro, CZ, Italy; s.bonacci@unicz.it (S.B.)
[4] National Council of Research, Institute of BioEconomy (CNR-IBE), Via Paolo Gaifami 18, Catania, Italy; lucia.sollima@cnr.it (L.S.); mariagrazia.melilli@cnr.it (M.G.M.)
* Correspondence: mariagrazia.melilli@cnr.it

Supplementary Material

Table S1. Retention time (min.), coefficient of determination (R^2) and linear regression model of external standards used for calibration.

Aminoacid	(RT min)	R^2	Linear Regression
L-Alanine	47.55	0.958	y=941627x-83.308
L-Leucine	48.97	0.976	$y=1*(10^6)x-61.269$
L-Methionine	51.13	0.961	y=993983x-10.114
L-Phenylalanine	52.51	0.952	$y=1*(10^6)x+15.772$
L-Cystine	55.06	0.988	$y=1*(10^6)x-59.647$
L-Histidine	55.45	0.960	$y=1*(10^6)x-160.23$
L-Lysine	55.24	0.988	$y=1*(10^6)x-59.647$
